# Investigating spillover of multidrug-resistant tuberculosis from a prison: a spatial and molecular epidemiological analysis

**DOI:** 10.1186/s12916-018-1111-x

**Published:** 2018-08-03

**Authors:** Joshua L. Warren, Louis Grandjean, David A. J. Moore, Anna Lithgow, Jorge Coronel, Patricia Sheen, Jonathan L. Zelner, Jason R. Andrews, Ted Cohen

**Affiliations:** 1 Department of Biostatistics, Yale University, New Haven, CT 06510 USA; 2 Paediatric Infectious Diseases, Section of Paediatrics, Department of Medicine, Imperial College, London, W2 1NY UK; 3 Laboratorio de Investigacion y Desarrollo, Universidad Peruana Cayetano Heredia, San Martin de Porres, Lima, Peru; 4 TB Centre and Department of Clinical Research, London School of Hygiene and Tropical Medicine, London, UK; 5 Department of Epidemiology, University of Michigan, Ann Arbor, MI 48109 USA; 6 Department of Medicine, Stanford University, Stanford, CA 94305 USA; 7 Department of Epidemiology of Microbial Diseases, Yale University, New Haven, CT 06510 USA

**Keywords:** Antibiotic resistance, Bayesian statistics, Spatial analysis, Spillover analysis, Transmission

## Abstract

**Background:**

Congregate settings may serve as institutional amplifiers of tuberculosis (TB) and multidrug-resistant tuberculosis (MDR-TB). We analyze spatial, epidemiological, and pathogen genetic data prospectively collected from neighborhoods surrounding a prison in Lima, Peru, where inmates experience a high risk of MDR-TB, to investigate the risk of spillover into the surrounding community.

**Methods:**

Using hierarchical Bayesian statistical modeling, we address three questions regarding the MDR-TB risk: (i) Does the excess risk observed among prisoners also extend outside the prison? (ii) If so, what is the magnitude, shape, and spatial range of this spillover effect? (iii) Is there evidence of additional transmission across the region?

**Results:**

The region of spillover risk extends for 5.47 km outside of the prison (95% credible interval: 1.38, 9.63 km). Within this spillover region, we find that nine of the 467 non-inmate patients (35 with MDR-TB) have MDR-TB strains that are genetic matches to strains collected from current inmates with MDR-TB, compared to seven out of 1080 patients (89 with MDR-TB) outside the spillover region (*p* values: 0.022 and 0.008). We also identify eight spatially aggregated genetic clusters of MDR-TB, four within the spillover region, consistent with local transmission among individuals living close to the prison.

**Conclusions:**

We demonstrate a clear prison spillover effect in this population, which suggests that interventions in the prison may have benefits that extend to the surrounding community.

**Electronic supplementary material:**

The online version of this article (10.1186/s12916-018-1111-x) contains supplementary material, which is available to authorized users.

## Background

In 2016, the latest year for which estimates are available, there were 490,000 incident cases of multidrug-resistant tuberculosis (MDR-TB) [1]. Individuals with MDR-TB have a disease that is resistant to at least isoniazid and rifampicin and they are at substantially elevated risk of treatment non-response, treatment-related side effects, and mortality, even if drug resistance is recognized and treatment with appropriate second-line drug regimens is available [2–4].

MDR-TB arises as a consequence of failed treatment or by direct transmission from an individual infectious with MDR-TB. Measures of the relative importance of failed treatment and direct transmission as drivers of MDR-TB are not easy to obtain in the setting of complex epidemics, where reports of treatment history and prior drug susceptibility results are often unreliable or unavailable. Nonetheless, an analysis based on programmatic data [5] and an inference based on fitting transmission dynamic models to data [6] reveal that direct transmission of MDR-TB is now the dominant mechanism driving incidence in most settings. Therefore, the success of interventions that aim to mitigate the rise of MDR-TB will depend critically on their ability to identify where transmission occurs and who is at the highest risk of infection.

It has been suggested that specific types of congregate settings, especially hospitals and prisons, can serve as institutional amplifiers of TB [7, 8], and in particular, MDR-TB [9–13]. This hypothesis suggests that the high incidence rates of TB and MDR-TB reported in congregate settings can lead to spillover risk in the community [14], especially in settings where there is a rapid turnover of members in the congregate setting or there are opportunities for interaction between community members and those in the congregate setting. Consistent with this hypothesis, a statistical analysis of country-level data from eastern Europe and central Asia found that rates of growth of the prison population were positively associated with increases in both TB incidence and the risk of MDR-TB [15]. Several studies have also documented the likely spillover of TB from prisons to communities [16] and an increased risk of MDR-TB in spatial proximity to prisons [12, 17] and in areas where former prisoners reside [18].

In this work, we develop hierarchical Bayesian statistical models to investigate the hypothesis that an elevated MDR-TB risk for prisoners (documented in an earlier study [19]) produces detectable spillover effects in the surrounding neighborhoods of Lima, Peru. In our analytic framework, we simultaneously test this hypothesis and estimate the magnitude, shape, and spatial range of the spillover effect. In addition, we further investigate the possibility of local transmission of MDR-TB within these neighborhoods through an analysis of the residual spatial correlation in risk among the patients and an exploration of genetic clusters of specific strains of *Mycobacterium tuberculosis*.

## Methods

### Data description

**Fig. 1:**
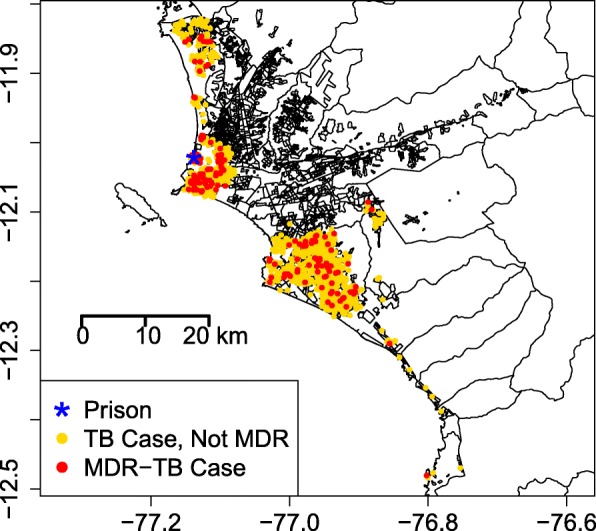
Graphical summary of the study population. Patient locations are jittered to protect confidentiality. Black lines represent within-region boundaries. MDR-TB Multidrug-resistant tuberculosis

Between 2008 and 2010, sputum, as well as basic demographic and clinical data, were collected from all individuals with suspected TB living in two of the four large regions of metropolitan Lima (Callao and Lima Sur). The geographic region and study population are presented in Fig. 1 (jittered to protect confidentiality). These data were collected in the context of a population-wide implementation study of the Microscopic Observation Drug Susceptibility assay, a rapid test for TB and MDR-TB. Full details of the field methods are available in a previous publication [19]. All isolates included in this study have been tested for susceptibility to isoniazid and rifampin and have been genotyped by 15-loci MIRU-VNTR [20]. In total, approximately 71% of all culture-positive isolates had genotyping and geographic data and were included in this analysis [19].

For this analysis, we used individual-level information about the patients including sex (male or female), sputum smear positivity indicator (yes or no), previous TB treatment status (yes or no), average socioeconomic status of their city block (lower, middle, and upper tertiles), population density of their city block (number of people per city block), age category (<25, 25–64, or 65+ years), prisoner status (yes or no), and longitude and latitude of residence at time of diagnosis. In total, our analysis includes 1587 TB patients after removing those with missing covariate information. Of these patients, 115 shared a residence with at least one other patient in the study. Table 1 displays the summary information for this population by MDR-TB status.

**Table 1:** Study population characteristics

	Tuberculosis type	
Characteristic	Multidrug-resistant	Drug susceptible
Total	164	1423
Prisoner status (yes)	7 (0.04)	33 (0.02)
Sex (male)	102 (0.62)	897 (0.63)
Smear positive (yes)	147 (0.90)	1271 (0.89)
Previous treatment (yes)	79 (0.48)	346 (0.24)
Socioeconomic status category		
Upper tertile	9 (0.05)	73 (0.05)
Middle tertile	65 (0.40)	485 (0.34)
Lower tertile	90 (0.55)	865 (0.61)
Age category		
[18–25)	36 (0.22)	376 (0.26)
[25–65)	120 (0.73)	951 (0.67)
65+	8 (0.05)	96 (0.07)
Population density (per city block)	127.99 (57.84)	121.90 (57.38)
Distance to prison (kilometers)	15.07 (12.10)	18.36 (11.57)

Counts with proportions in parentheses are shown for categorical variables. Means with standard deviations in parentheses are shown for continuous variables

### Spillover risk analysis

We develop hierarchical Bayesian statistical models that simultaneously account for the potential of elevated MDR-TB risk for an individual due to a number of sources including (i) individual-level risk factors, (ii) proximity to the prison (representing potential spillover), and (iii) spatial proximity to other MDR-TB cases (representing the possibility of local transmission). In our analyses, each TB patient is categorized as having MDR-TB or drug-susceptible TB (i.e., any phenotype that is not MDR-TB) and we model the probability that a patient has MDR-TB as a function of these different sources of risk.

Specifically, we define *Y*_*i*_(***s***_*i*_) ∣ *p*_*i*_(***s***_*i*_)~Bernoulli(*p*_*i*_(***s***_*i*_)), *i* = 1, …, *n*, where *Y*_*i*_(***s***_*i*_) is equal to 1 if individual *i* residing at spatial location ***s***_*i*_ has MDR-TB and is equal to 0 otherwise. *p*_*i*_(***s***_*i*_) describes the individual’s personal probability of being an MDR-TB patient and *n* is the number of individuals in the study. We note that multiple individuals can be located at the same residence, leading to identical spatial locations in the analysis. Therefore, we define the set of unique spatial locations as ${\boldsymbol{s}}_j^{\ast}$. Each ***s***_*i*_ maps to a particular ${\boldsymbol{s}}_j^{\ast}$ for *j* = 1, …*m*, where *m* represents the total number of unique spatial locations and is less than the total number of patients, *n*.

Next, we introduce a model for an individual’s personal probability of having MDR-TB that accounts for the patient’s personal risk factors, distance to the prison, and spatial proximity to other individuals such that

$$ {\Phi}^{-1}\left({p}_i\left({\boldsymbol{s}}_i\right)\right)={\mathbf{x}}_i^T\boldsymbol{\beta} +\lambda g\left(\left\Vert {\boldsymbol{s}}_i-{\boldsymbol{s}}_p\right\Vert; \theta \right)+w\left({\boldsymbol{s}}_i\right), $$

where Φ^−1^(.) is the inverse cumulative distribution function of the standard normal distribution, resulting in a probit regression model. **x**_*i*_ is a vector of individual-level risk factors, which are displayed in Table 2. ***β*** is a vector of unknown regression parameters. The function *λg*(‖***s***_*i*_ − ***s***_*p*_‖; *θ*) describes the impact of a patient’s proximity to the prison on MDR-TB risk, where ***s***_*p*_ is the longitude and latitude of the prison, ‖.‖ is the Euclidean distance function, and *λ*, *θ* are unknown parameters that describe the magnitude of the spillover risk and the spatial range of the spillover effect, respectively. Finally, *w*(***s***_*i*_) is a spatially correlated random effect specific to the individual’s location of residence that is useful in identifying residual MDR-TB risk based on spatial location alone, which is risk that is potentially due to local transmission.

**Table 2:** Inference from the Gaussian spillover risk model

			Quantile	
Parameter	Mean	SD	0.025	0.975
Intercept	–2.23	0.71	–3.90	–1.20
Previous treatment: yes vs. no	0.81	0.24	0.44	1.35
Sex: female vs. male	0.11	0.16	–0.17	0.46
Smear positive: yes vs. no	0.11	0.22	–0.29	0.58
Socioeconomic status:				
Middle vs. upper	–0.19	0.30	–0.81	0.39
Lower vs. upper	–0.40	0.31	–1.10	0.15
Population density	0.01	0.09	–0.17	0.19
Age category				
[25–65) vs. [18–25)	–0.01	0.16	–0.33	0.31
65+ vs. [18–25)	–0.27	0.32	–1.00	0.30
Spillover magnitude (*λ*)	0.49	0.28	0.01	1.13
Spillover range (*θ*), kilometers	5.47	1.83	1.38	9.63
Regression parameter variance (${\sigma}_{\delta}^2$)	0.90	0.86	0.18	3.10
Spatial variance parameter (${\sigma}_w^2$)	1.71	1.55	0.11	5.53

Posterior means, posterior SDs, and posterior quantiles are presented. Parameters whose 95% credible intervals do not include 0 are shown in bold, indicating an increased (positive effect) MDR-TB risk for a patient with the particular characteristic

*MDR-TB* multidrug-resistant tuberculosis, *SD* standard deviation

We are primarily interested in determining if proximity to the prison has any impact on an individual’s MDR-TB risk and formally test this hypothesis through the inclusion of *λg*(‖***s***_*i*_ − ***s***_*p*_‖; *θ*). We test a number of competing options that each make a different assumption regarding the range and shape of the potential spillover effect, and formally compare the models using two Bayesian model selection techniques: the Watanabe–Akaike information criterion (WAIC) [21, 22] and *D*_*k*_ [23]. WAIC is used primarily when the model is intended for explanatory purposes while *D*_*k*_, a posterior predictive loss metric, is used to compare the predictive capabilities of different models. Both metrics balance model fit and complexity with smaller values of each being preferred. Following [24], we set *k* = 10^10^ and use the Bernoulli distribution deviance, with continuity correction, when calculating *D*_*k*_. Our competing models are created by defining *g*(‖***s***_*i*_ − ***s***_*p*_‖; *θ*) as 1(‖***s***_*i*_ − ***s***_*p*_‖ = 0) (prisoner indicator), 1(‖***s***_*i*_ − ***s***_*p*_‖ ≤ *θ*) (constant spillover risk), exp{−‖***s***_*i*_ − ***s***_*p*_‖}1(‖***s***_*i*_ − ***s***_*p*_‖ ≤ *θ*) (exponential spillover risk), and exp{−‖***s***_*i*_ − ***s***_*p*_‖^2^}1(‖***s***_*i*_ − ***s***_*p*_‖ ≤ *θ*) (Gaussian spillover risk), where 1(.) is an indicator function that is equal to 1 if the input statement is true and is equal to 0 otherwise.

The prison indicator model assumes that only those patients located at the prison have increased MDR-TB risk, indicating no spillover effect. The constant spillover risk model suggests that there is a spillover effect extending outside the prison that is constant in magnitude for all patients within the range of influence (controlled by the unknown parameter *θ*). The exponential spillover risk model suggests that the risk is highest at the prison and decays based on the function exp{−‖***s***_*i*_ − ***s***_*p*_‖}1(‖***s***_*i*_ − ***s***_*p*_‖ ≤ *θ*) as distance from the prison increases. After a certain distance *θ*, the risk is, again, assumed to be zero. The Gaussian spillover risk model is similar to the exponential version, except that it replaces the exponential decay function with exp{−‖***s***_*i*_ − ***s***_*p*_‖^2^}1(‖***s***_*i*_ − ***s***_*p*_‖ ≤ *θ*).

We are also interested in understanding if there is additional residual risk associated with proximity to other MDR-TB cases. Therefore, we introduce random effects that aim to detect pockets of increased MDR-TB risk due to spatial location alone. The *w*(***s***_*i*_) parameters are spatially correlated random effects that account for any residual spatial variability in MDR-TB risk (after controlling for individual-level characteristics and proximity to the prison). The vector of spatially correlated random effects, $\boldsymbol{w}={\left\{w\left({\boldsymbol{s}}_1^{\ast}\right),\dots, w\left({\boldsymbol{s}}_m^{\ast}\right)\right\}}^T$, is modeled using a Gaussian process prior distribution with spatially structured covariance matrix [25] such that $\boldsymbol{w}\mid \phi \sim \mathrm{MVN}\left(\mathbf{0},{\sigma}_w^2\Sigma \left(\phi \right)\right)$ where MVN(., .) represents the multivariate normal distribution and ${\sigma}_w^2\Sigma \left(\phi \right)$ describes the variance/covariance of the random effects. This specification allows us to determine if there are highly localized regions of MDR-TB risk, possibly due to transmission. Random effects associated with individuals who are separated by a short distance are assumed to be more similar a priori, leading to similar estimates of individual-level risk (*p*_*i*_(***s***_*i*_)). We allow the data to inform about the distance that this correlation extends from a particular location and what type of impact it has on MDR-TB risk in general. Specifically, we model the covariance between two of the random effects by defining ${\sigma}_w^2\Sigma {\left(\phi \right)}_{ij}$ as

$$ \mathrm{Covariance}\left\{w\left({\boldsymbol{s}}_i^{\ast}\right),w\left({\boldsymbol{s}}_j^{\ast}\right)\right\}={\sigma}_w^2\rho \left(\left\Vert {\boldsymbol{s}}_i^{\ast }-{\boldsymbol{s}}_j^{\ast}\right\Vert; \phi \right), $$

where ${\sigma}_w^2$ represents the total variance of the random effect distribution, *ϕ* controls the range of spatial correlation (at what distance random effects are uncorrelated), and *ρ*(.; .) is an isotropic spatial correlation function that describes the correlation between random effects as a function of the distance between spatial locations [25]. In our application of the model, we choose the spherical correlation structure because it provides us with an exact definition of the range of spatial correlation, 1/*ϕ*. The spherical correlation function is defined as

$$ \rho \left(d;\phi \right)=\left\{\begin{array}{c}1-1.5\phi d+0.5{\left(\phi d\right)}^3,\kern0.5em \mathrm{if}\ 0\le d\le 1/\phi, \\ {}0,\kern0.5em \mathrm{if}\ d\ge 1/\phi, \end{array}\right. $$

where *d* is the distance between spatial locations.

Predicted probabilities of MDR-TB at new spatial locations are obtained through the posterior predictive distribution of individual-level probabilities, *f*(*p*_*i*_(***s***_*i*_)| ***Y***), where ***Y*** = {*Y*_1_(***s***_1_), …, *Y*_*n*_(***s***_*n*_)}^*T*^, using properties of the conditional multivariate normal distribution and composition sampling [25]. The mean and standard deviation of the posterior predictive distributions are plotted to assess the geographic risk of MDR-TB across the study region.

### Molecular analysis

The spatially correlated random effects identify areas that have excess residual MDR-TB risk. To determine if this excess risk may be due to local transmission, we further interrogate these regions using 15-loci MIRU-VNTR genotypes [20]. If multiple genetically matched isolates are identified in a single high MDR-TB risk region, we deem local transmission to be probable. Specifically, we first identify estimated spatial random effects whose upper 95% credible intervals are larger than 0, indicating a statistically significant increased local risk of MDR-TB (i.e., $P\left(w\left({\boldsymbol{s}}_j^{\ast}\right)>0|\boldsymbol{Y}\right)\ge 0.95$). Next, based on the estimated spatial range of correlation for these random effects (posterior mean of 1/*ϕ*), we create buffers around these significant spatial random effects with a radius equal to this distance. We then look within these buffers to determine if there are at least two individuals with a statistically significant increased MDR-TB risk. For those buffers that meet these requirements, we examine whether the observed strains have identical MIRU-VNTR patterns.

We also examine the MDR-TB strains from individuals residing within the estimated range of the spillover effect from the prison (posterior mean of *θ*). These MDR-TB strains are then compared with MDR-TB strains from current inmates to investigate further the possible mechanism of the spillover effect identified through the spatial analysis.

### Prior specification

To specify the model fully within the Bayesian framework, prior distributions must be selected for each of the unknown model parameters. When possible, we select weakly informative prior distributions for the data to drive the inference rather than our prior beliefs. The regression parameters are assumed to arise independently from a common Gaussian distribution such that ${\beta}_j,\lambda \sim \mathrm{N}\left(0,{\sigma}_{\delta}^2\right)$ with ${\sigma}_{\delta}^2\sim \mathrm{Inverse}\ \mathrm{Gamma}\left(0.01,0.01\right)$. The spillover range parameter, *θ*, is assigned a Uniform(0, 10) kilometers prior based on the distribution of patients surrounding the prison and reasonable expectations regarding the distance of a spillover impact. The variance of the spatial random effect distribution, ${\sigma}_w^2$, is given an Inverse Gamma(0.01, 0.01) prior while a Gamma(0.10, 0.10) prior distribution is selected for the spherical correlation range parameter, *ϕ*. In addition, we assess the sensitivity of our results to the choice of prior distributions for the variance parameters by rerunning the final selected model while specifying *σ*_*δ*_, *σ*_*w*_~Uniform(0, 100).

### Computing and model fitting

Each of the proposed models are fitted in the Bayesian setting using Markov chain Monte Carlo sampling techniques with R statistical software [26]. For each model, we collect 90,000 samples from the joint posterior distribution of the model parameters after a burn-in period of 10,000 iterations. To reduce the autocorrelation in the Markov chains and ease the computational burden of summarizing 90,000 posterior samples (particularly with respect to prediction), we thin the chains, resulting in a final set of 5000 posterior samples. Convergence was assessed through visual inspection of individual parameter trace plots and by monitoring the Geweke diagnostic measure [27]. Neither approach suggested any obvious signs of non-convergence.

## Results

### Data description

We have a total of *n* = 1,587 TB patients in *m* = 1,509 unique spatial locations. As shown in Table 1, 164 of the TB patients have MDR-TB (10.3%). The factor most closely associated with increased risk of MDR-TB is previous treatment for TB; 18.6% of previously treated individuals have MDR-TB compared to 7.3% of treatment naive individuals. We note that previous TB treatment status among those with MDR-TB is an imperfect proxy for transmitted MDR-TB. Individuals without previous treatment are assumed to have MDR-TB as a consequence of direct transmission, but those with previous treatment may have MDR-TB as a result of transmission or acquisition during their prior treatment. Current imprisonment is also associated with MDR-TB. Among the 40 inmates with TB, 17.5% have MDR-TB compared to 10.2% of individuals in the general population.

### Spillover risk analysis

Additional file 1: Table S1 displays the model comparison results along with a measure of model complexity for each metric (*p*_WAIC_ for WAIC and *P* for *D*_*k*_). The prisoner indicator model provides an improved fit over the constant spillover risk model, indicating that the assumption of constant risk in the area surrounding the prison may not accurately reflect the true nature of the spillover. However, a substantial improvement in model fit is observed when different shapes of spillover risk are considered. The exponential and Gaussian spillover risk models have an improved fit overall compared with the prisoner indicator model. This indicates that there may be a spillover effect and that the resulting excess risk decreases as distance from the prison increases, before becoming 0.

The WAIC and *D*_*k*_ results between these two models are comparable overall, so we examine the inference for *λ*, the parameter controlling the magnitude of the spillover risk, to make our final model selection. While the posterior mean of *λ* is comparable between both models, the 95% credible interval of the parameter for the exponential spillover risk model is slightly below 0. The corresponding interval from the Gaussian spillover risk model excludes 0 (Table 2). Therefore, we further explore the results of the Gaussian spillover risk model in the remaining analyses but note that the results are generally comparable between both models.

In Table 2, we present the posterior inference for each of the parameters in the Gaussian spillover risk model. Parameters whose 95% credible intervals are strictly larger than 0 indicate an increased risk of MDR-TB for patients in those categories, with a similar interpretation for strictly negative results. As expected, patients who have been previously treated for TB are more likely to have MDR-TB than patients with no previous treatment history. No other individual-level risk factors are associated with increased or decreased risk of MDR-TB.

**Fig. 2:**
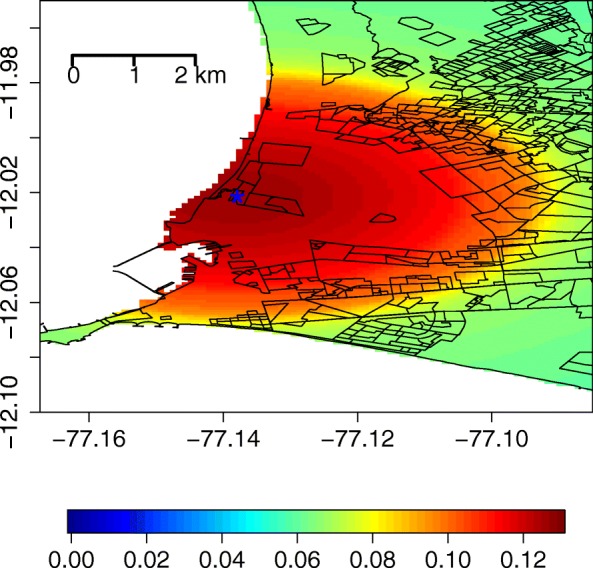
MDR-TB spillover risk predictions. Predicted probability of MDR-TB due only to the estimated prison spillover effect for a patient with previous TB treatment in the Gaussian spillover model. MDR-TB Multidrug-resistant tuberculosis

Inference for *λ* in Table 2 suggests that people living closer to the prison are at a higher risk of MDR-TB. The spatial range of the spillover effect, described by *θ*, is estimated to be 5.47 km, indicating that the increased risk extends beyond the prisoner population. The prior and posterior densities for *λ* and *θ* are shown in Additional file 1: Figures S1 and S2, respectively. Inside this spillover region, 14.8% of patients have MDR-TB while outside the spillover region the risk is only 8.2%. In Fig. 2, we display the predicted probability of MDR-TB across the region for a patient with previously treated TB while in Additional file 1: Figure S4, we display the predictions for a patient without previous TB treatment. We do not include the spatial random effects when calculating these probabilities to focus attention solely on the spillover risk. These figures clearly show the elevated MDR-TB risk surrounding the prison, the decay in risk as distance from the prison increases, and the large difference in risk between patients with and without a history of previous TB treatment. Posterior standard deviations for these plots are shown in Additional file 1: Figures S3 and S4.

### Molecular analysis

Through incorporation of the MIRU-VNTR genotyping data, we also investigate the particular TB strains that are present within the estimated buffer of increased MDR-TB risk surrounding the prison. In total, there are 467 non-prisoner TB patients within 5.47 km (posterior mean of *θ*) of the prison. Of the TB strains observed in this spillover region, 249 (49%) do not have an exact MIRU-VNTR match. Nine MDR-TB patients outside the prison (but within the spillover buffer) share a common strain with an inmate with MDR-TB. In contrast, outside this prison spillover buffer, where there are over twice as many TB patients (1080), only seven MDR-TB patients share a common strain with inmates with MDR-TB (*p* = 0.022 from a two-sample test of proportions). When subsetting to only those patients with MDR-TB, we find nine out of the 35 MDR-TB patients within the prison spillover buffer share a common strain with an inmate compared to seven out of 89 MDR-TB patients outside the prison spillover buffer (*p* = 0.008). This provides further evidence to support the idea of potential MDR-TB spillover from the prison.

Estimation of the spherical correlation range parameter, *ϕ*, suggests that the residual spatial correlation has a highly localized impact (0.13 km, 95% credible interval: 0.04, 0.28 km). Individuals separated by distances greater than this are essentially independent of each other with respect to residual MDR-TB risk. Individuals living within this distance have a more similar risk of MDR-TB, based on their proximity to each other alone. In total, 18 out of the *m* = 1,509 unique spatial location random effects have an upper 95% credible interval larger than zero. From these significant random effects, we identified eight unique spatial clusters of at least two patients with increased residual MDR-TB risk, four of these clusters within the prison buffer. Full information on each cluster is presented in Additional file 1: Table S2.

**Fig. 3:**
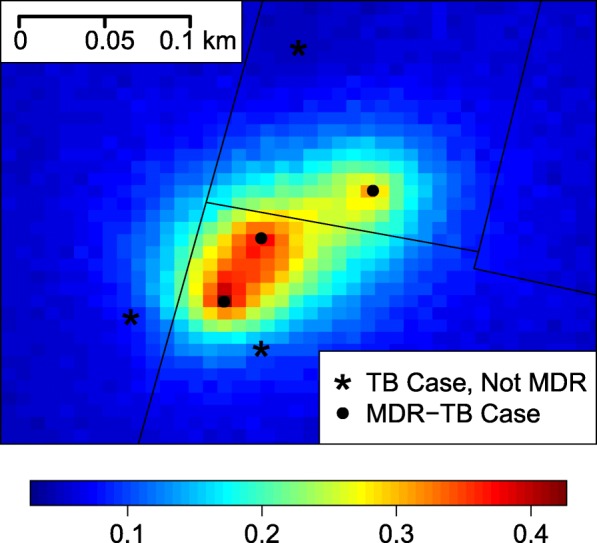
MDR-TB residual risk predictions. Predicted probability of MDR-TB for a patient without previous TB treatment in the Gaussian spillover model. Note that two MDR-TB patients are co-located. MDR-TB Multidrug-resistant tuberculosis

As an example of the role of residual spatial variability in local MDR-TB risk in this region, in Fig. 3 we display a cluster of four patients and the predicted risk of MDR-TB in the area assuming a patient had not been previously treated for TB (none of these patients had been previously treated). The posterior standard deviations are presented in Additional file 1: Figure S5. The elevated risk in this localized area, due to the inclusion of the spatial random effects, strongly suggests local transmission. In this cluster, where two of the patients were co-located, three of them share the same TB genotype. Interestingly, the two co-located patients do not match with respect to TB genotype, a phenomenon we have also seen in previous household studies of MDR-TB in Lima [28].

When investigating the robustness of our findings to the choice of prior distributions for the variance parameters, the sensitivity analysis results suggest that estimation of the spatial range of the spillover effect (5.29 vs. 5.47 km) and of the residual spatial correlation (0.11 vs. 0.13 km) were similar. Therefore, the estimated impact of the prison location and of potential local transmission on MDR-TB risk in the community remains consistent across the different sets of prior distributions.

## Discussion

The availability of spatial and pathogen genetic data offers new opportunities to describe the transmission dynamics of pathogens across spatial scales [29], and these types of data have been combined to gain a better understanding of how MDR-TB is transmitted within cities [30] and over larger geographic areas [18, 31], but the role of prisons in propagating epidemics of MDR-TB in the community has not previously been confirmed.

In this study, we found that the risk of MDR-TB was elevated among individuals diagnosed with TB in the area surrounding the prison in Lima. This spillover effect dissipated as distance from the prison increased, and the effect was non-significant at a distance of approximately 5 km. The individual covariate known to be most associated with MDR-TB (i.e., previous treatment for TB) remained a significant risk factor, but the distribution of cases reporting previous treatment did not explain the spatial concentration of MDR-TB around the prison location. As there is little reason to believe that risk of acquired resistance should be related to proximity to the prison, this spatial pattern suggests that the majority of MDR-TB cases among previously treated individuals in this area may be the result of transmitted resistance. Our approach allowed us to identify foci of residual risk of MDR-TB, for which interrogation of molecular epidemiological data revealed several probable hot spots of MDR-TB transmission with strains that are were also found within the prison. In summary, our analysis suggests that those living in the area closest to the prison experience a higher risk of MDR-TB spillover, and once such strains appear outside the prison, they can be transmitted further in the community. Demonstrating a clear prison spillover effect highlights the need to intervene in the prison to prevent both internal and external TB transmission. Figures from the Peruvian National Penitentiary Institute demonstrate that Sarita Colonia prison in Callao is overpopulated by 483%. The prison was designed to have a capacity of 573 inmates but in October 2016 it had a prison population of 3332 [32]. Daily mixing between the prison population and the surrounding community occurs because of the flux of prison staff and visitors, which includes conjugal and intimate visits, prisoners with permission to leave, and the continual intake of new inmates and the release of inmates. These types of movements provide a potential explanation for how the risk of MDR-TB can extend beyond the walls of the prison [33].

Our study has several notable limitations. First, we do not have data on whether individuals with TB in the community had previously been imprisoned or had known exposure to prisoners or ex-prisoners. This would have been useful in understanding the mechanism of increased risk experienced by those living closest to the prison. Second, our analysis is based solely on household location. As transmission of *Mycobacterium tuberculosis* may well occur outside the home, use of home location serves at best as a proxy of transmission risk. Third, we had sufficient data to include 71% of culture-positive isolates in this analysis, and it is possible that selection bias could occur if individuals without bacteriological confirmation of TB or missing drug susceptibility testing or spatial data were at a systematically different risk of MDR-TB than those included in the analysis. Fourth, we have used MIRU-VNTR data to identify strains that are genetically clustered and thus, may be related in chains of transmission. While MIRU-VNTR is an important tool for identifying potential transmission clusters, whole-genome sequencing can break up apparent MIRU-VNTR clusters [34] and may have allowed us to infer transmission events better. [35] We are hopeful that future work, in which whole-genome sequencing is combined with spatial and epidemiological data to pin down the role of specific institutions in the propagation of TB epidemics, will inform the targeting of transmission-blocking interventions to settings where they can have the greatest effect. Finally, it is possible that ecological bias may be introduced by analyzing individual-level data using a combination of individual- and city block-level covariates. Associations could potentially differ if all covariates were measured on the same spatial scale.

## Conclusions

We leveraged epidemiological, spatial, and pathogen genetic data to test the hypothesis that high rates of MDR-TB previously documented within a prison have led to a spillover risk in the surrounding community. Using Bayesian hierarchical spatial statistical modeling, we found strong evidence to support the hypothesis that the excess risk extends beyond the walls of the prison.

In combination with existing work, our results suggest that such institutions have potential to amplify epidemics and that efforts to control transmission within institutions can also have important indirect effects on reducing risk in the surrounding community.

## Additional file

Additional file 1: Additional tables and figures. **Table S1.** Model comparison results with smaller values of WAIC and *D*_*k*_ preferred. **Table S2.** Residual MDR-TB risk spatial cluster results. **Figure S1.** *θ* prior density (dashed line) and posterior histogram plots. **Figure S2.** *λ* prior density (dashed line) and posterior histogram plots. **Figure S3.** Posterior standard deviations for predictions presented in Fig. 2 of the main text. **Figure S4.** MDR-TB spillover risk predictions and uncertainty. **Figure S5.** Posterior standard deviations for predictions presented in Fig. 3 of the main text. (DOCX 393 kb)

## Abbreviations

MDR-TB: Multidrug-resistant tuberculosis
SD: Standard deviation
TB: Tuberculosis
WAIC: Watanabe–Akaike information criterion
