# Supplementary material for: Investigating spillover of multidrug-resistant tuberculosis from a prison: a spatial and molecular epidemiological analysis
Source: BMC Med. 2018 Aug 3;16:122. doi: 10.1186/s12916-018-1111-x (PMC6091024; doi:10.1186/s12916-018-1111-x)

**Additional file 1**

**Title:** Investigating spillover of multidrug-resistant tuberculosis from a prison: a spatial and molecular epidemiological analysis

**Authors:** Joshua L. Warren^1^, Louis Grandjean^2,3^, David A.J. Moore^3,4^, Anna Lithgow^4^, Jorge Coronel^3^, Patricia Sheen^3^, Jonathan L. Zelner^5^, Jason R. Andrews^6^, Ted Cohen^7^

**Email addresses:** [joshua.warren@yale.edu](mailto:joshua.warren@yale.edu), [lgrandjean@gmail.com](mailto:lgrandjean@gmail.com), [David.Moore@lshtm.ac.uk](mailto:David.Moore@lshtm.ac.uk), [lithgow.anna@gmail.com](mailto:lithgow.anna@gmail.com), [jcoronelh@yahoo.es](mailto:jcoronelh@yahoo.es), [patricia.sheen@upch.pe](mailto:patricia.sheen@upch.pe), [jzelner@umich.edu](mailto:jzelner@umich.edu), [jasonandr@gmail.com](mailto:jasonandr@gmail.com), [theodore.cohen@yale.edu](mailto:theodore.cohen@yale.edu).

**Institutional affiliations:** ^1^Department of Biostatistics, Yale University, New Haven, CT 06510; ^2^Paediatric Infectious Diseases, Section of Paediatrics, Department of Medicine, Imperial College, London, United Kingdom, W2 1NY; ^3^Laboratorio de Investigacion y Desarrollo, Universidad Peruana Cayetano Heredia, San Martin de Porres, Lima, Peru; ^4^TB Centre and Department of Clinical Research, London School of Hygiene and Tropical Medicine, London, United Kingdom; ^5^Department of Epidemiology, University of Michigan, Ann Arbor, MI 48109; ^6^Department of Medicine, Stanford University, Stanford, CA 94305; ^7^Department of Epidemiology of Microbial Diseases, Yale University, New Haven, CT 06510

**Corresponding author:** Joshua L. Warren, Ph.D., Department of Biostatistics, Yale University, Laboratory of Epidemiology and Public Health, 60 College St, New Haven, CT 06510. E-mail: [joshua.warren@yale.edu](mailto:joshua.warren@yale.edu); Phone: (203) 785-4188.

**Additional tables and figures:**

| Table S1: Model comparison results with smaller values of WAIC and *D_k_* preferred. * indicates that the WAIC values are not significantly different at the $\alpha=0.05$ significance level. | | |
| --- | --- | --- |
| **Model** | **WAIC (**$\boldsymbol{p}_{\mathbf{WAIC}}$**)** | ***D_k_* (*P*)** |
| Prisoner Indicator | 941.45 (174.56) | 477.63 (289.93) |
| Constant Spillover Risk | 946.80 (168.54) | 486.02 (291.22) |
| Exponential Spillover Risk* | 908.59 (185.33) | 446.50 (280.26) |
| Gaussian Spillover Risk* | 909.66 (185.09) | 447.67 (280.35) |

| Table S2: Residual MDR-TB risk spatial cluster results. * indicates that the cluster is found within the estimated prison spillover range. “Distance” represents the average distance between patients in the cluster. | | | | |
| --- | --- | --- | --- | --- |
| **Cluster** | **Patients (MDR)** | **Location(s)** | **Strain(s)** | **Distance (km)** |
| 1* | 40 (7) | 1 | 17 | 0.00 |
| 2 | 4 (4) | 3 | 2 | 0.07 |
| 3 | 2 (2) | 2 | 1 | 0.02 |
| 4* | 2 (2) | 1 | 1 | 0.00 |
| 5 | 2 (2) | 2 | 2 | 0.01 |
| 6* | 2 (2) | 1 | 1 | 0.00 |
| 7* | 2 (2) | 1 | 1 | 0.00 |
| 8 | 2 (2) | 2 | 2 | 0.08 |

Figure S1: $\theta$ prior density (dashed line) and posterior histogram plots.


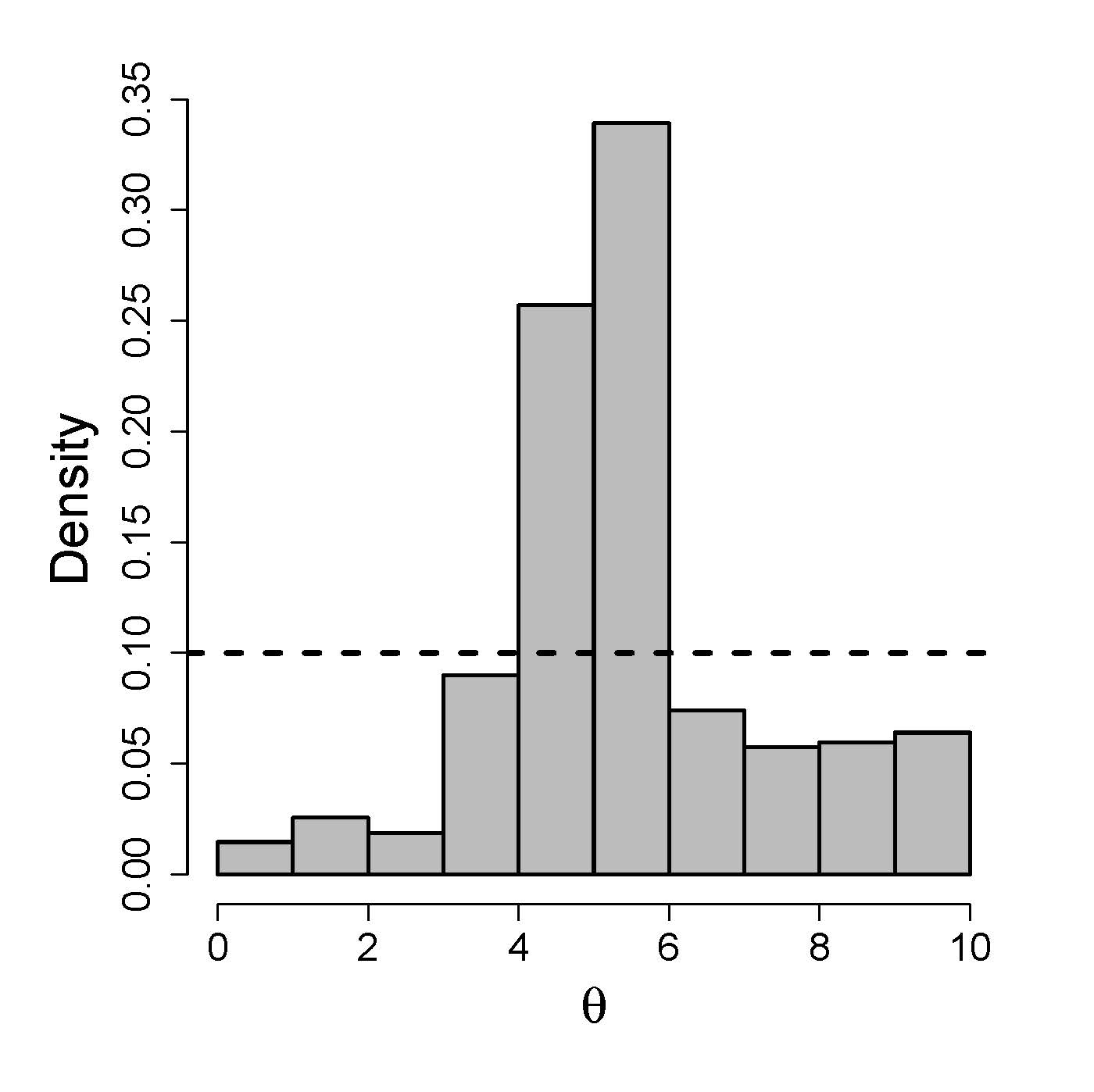


Figure S2: $\lambda$ prior density (dashed line) and posterior histogram plots.


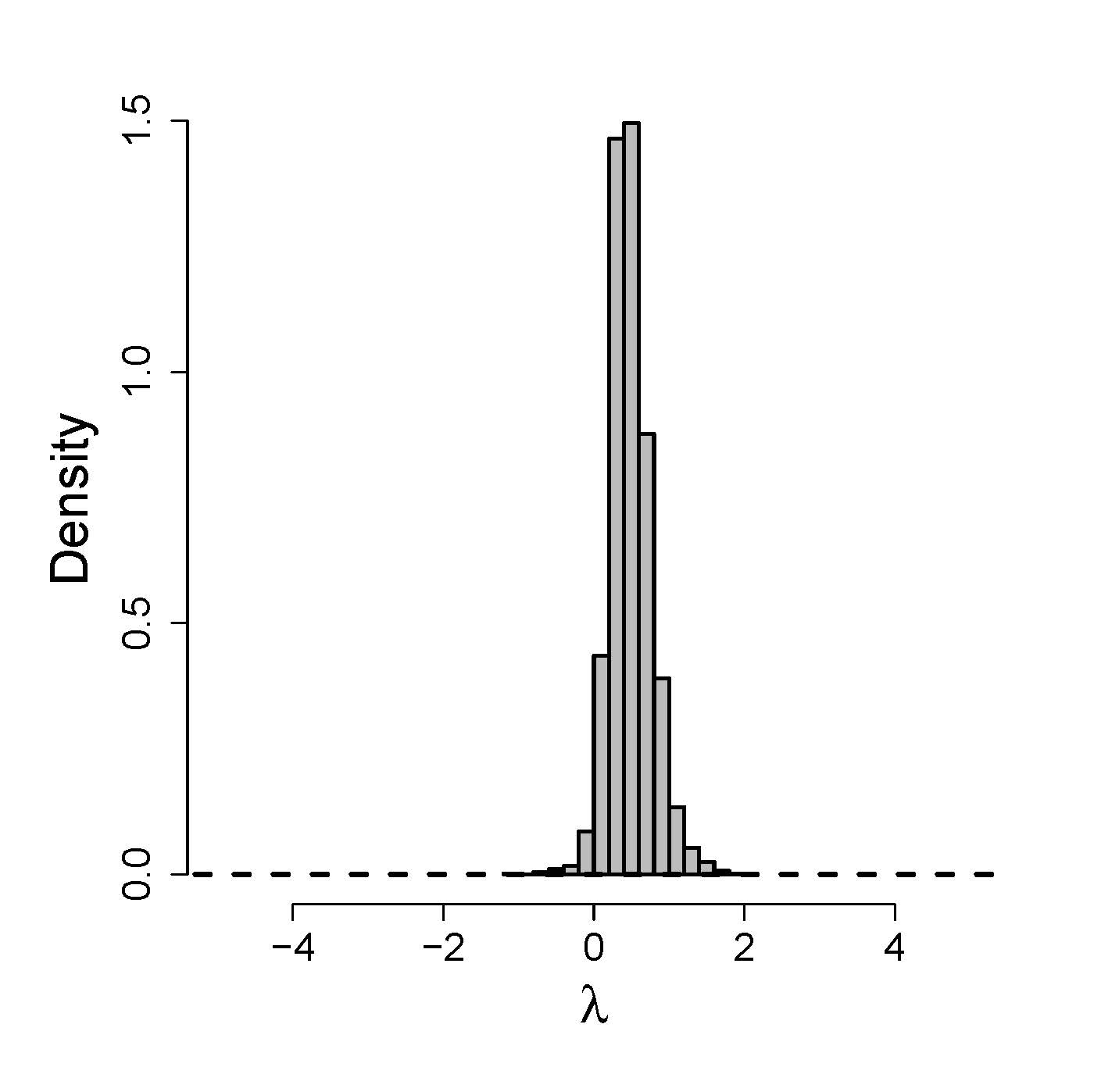


Figure S3: Posterior standard deviations for predictions presented in Figure 2 of the main text.


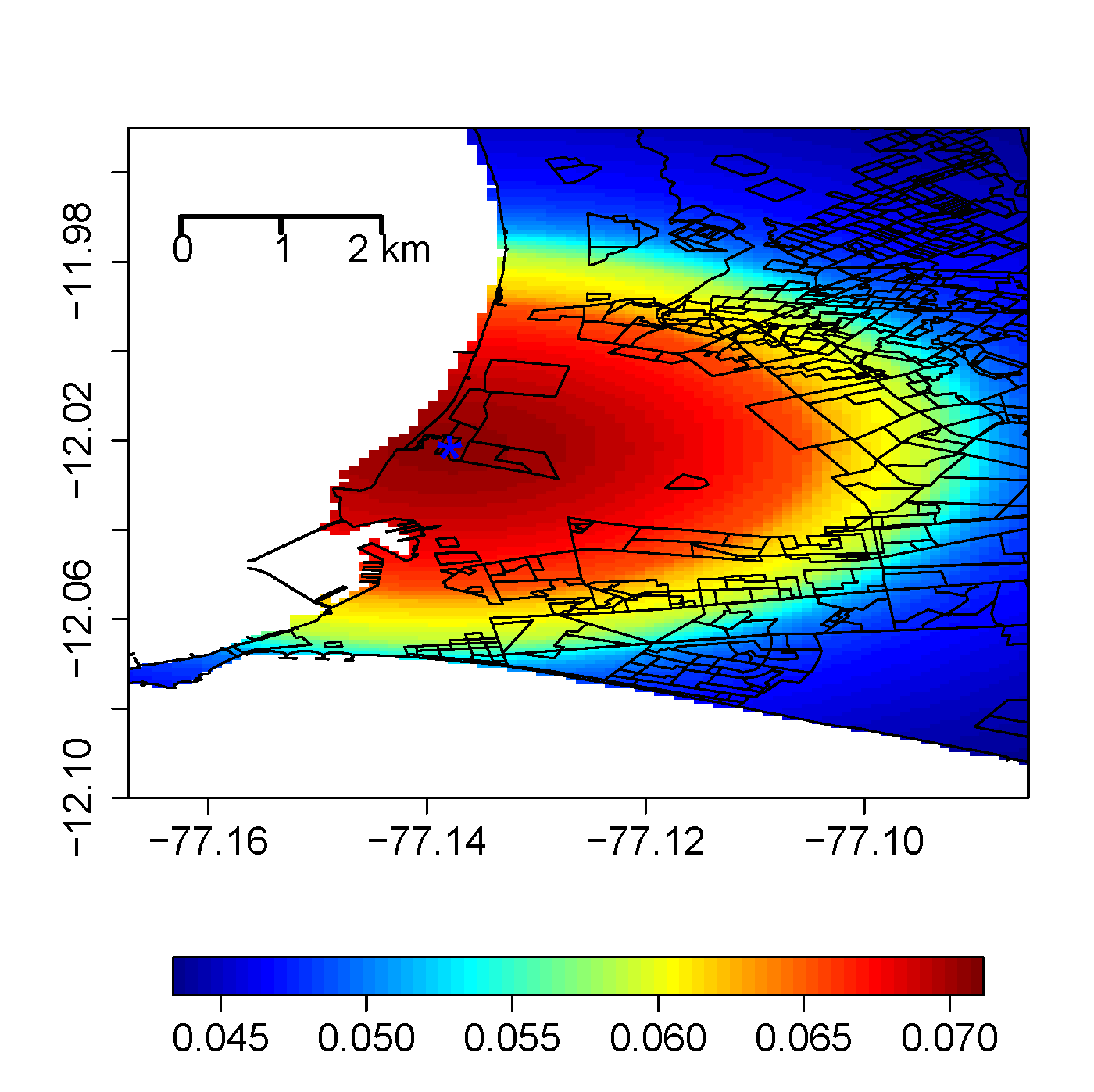


Figure S4: MDR-TB spillover risk predictions and uncertainty. Predicted probability of MDR-TB due only to the estimated prison spillover effect for a patient without previous TB treatment (left panel) and posterior standard deviations (right panel) in the Gaussian spillover model.


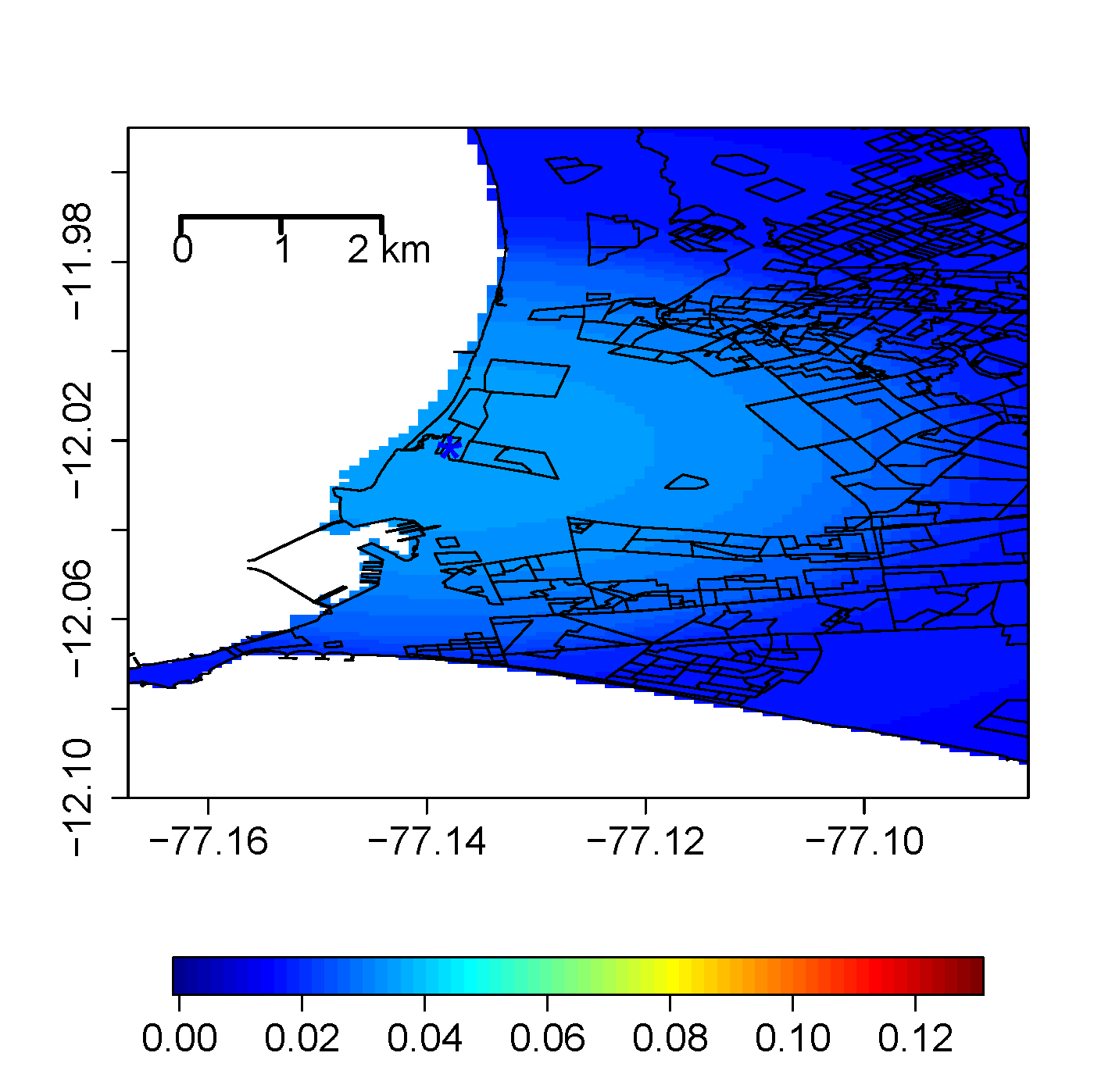

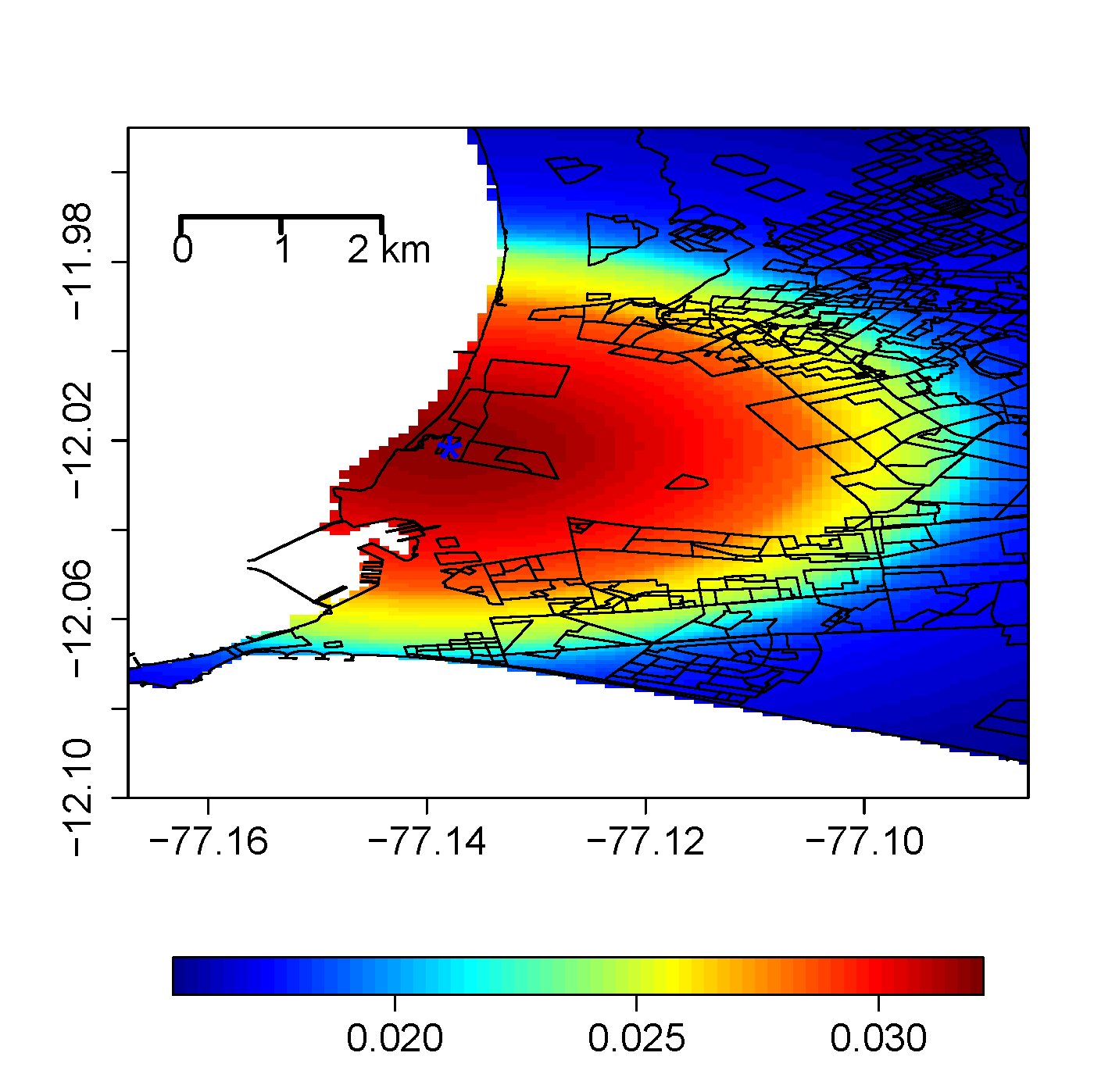


Figure S5: Posterior standard deviations for predictions presented in Figure 3 of the main text.


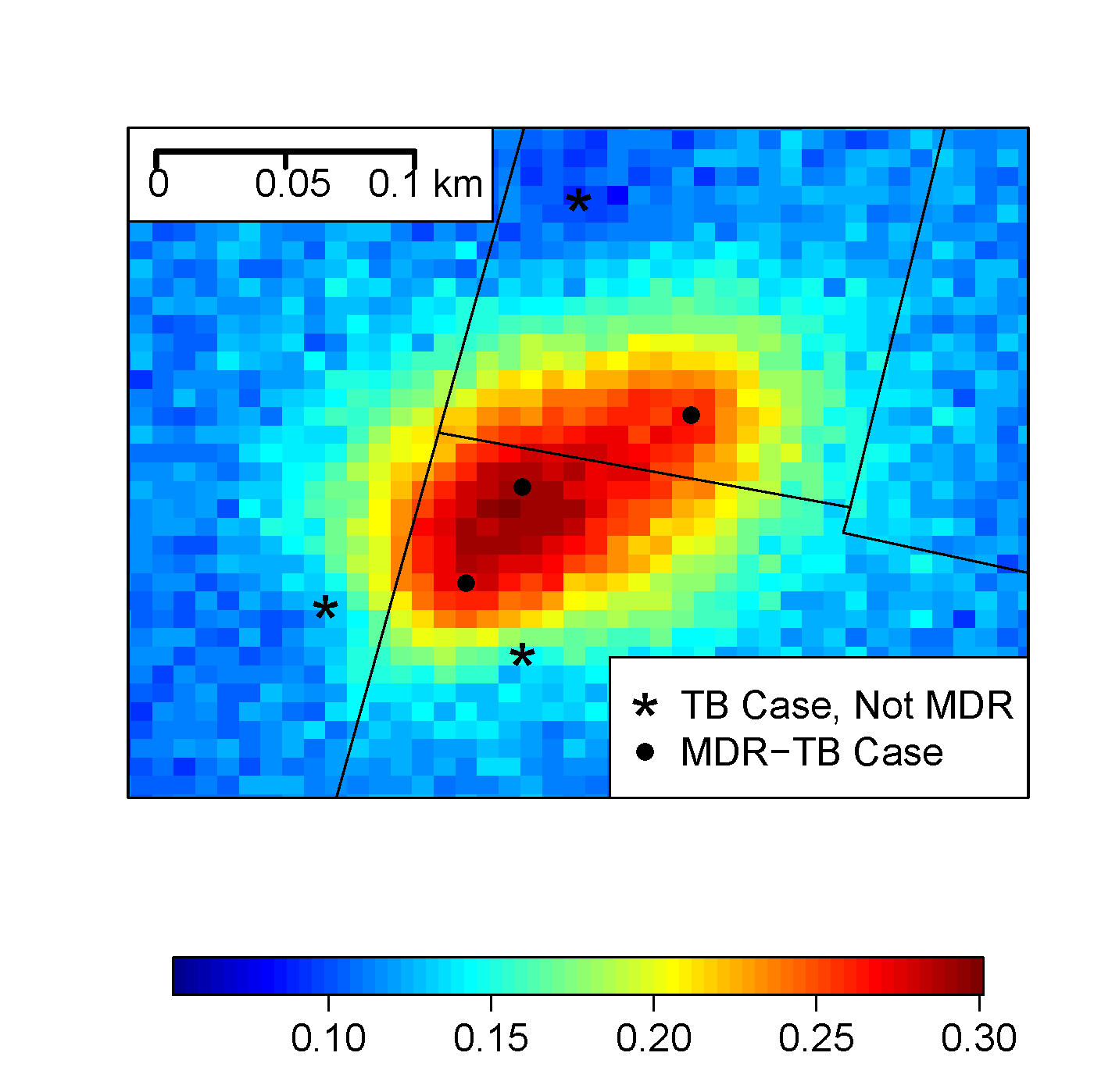

Supplement: Supplementary file 1 — Additional tables and figures. Table S1. Model comparison results with smaller values of WAIC and Dk preferred. Table S2. Residual MDR-TB risk spatial cluster results. Figure S1. θ prior density (dashed line) and posterior histogram plots. Figure S2. λ prior density (dashed line) and posterior histogram plots. Figure S3. Posterior standard deviations for predictions presented in Fig. 2 of the main text. Figure S4. MDR-TB spillover risk predictions and uncertainty. Figure S5. Posterior standard deviations for predictions presented in Fig. 3 of the main text. (DOCX 393 kb) [file 12916_2018_1111_MOESM1_ESM.docx]
